# Evaluation of Formal and Informal Spatial Coastal Area Planning Process in Baltic Sea Region

**DOI:** 10.3390/ijerph18094895

**Published:** 2021-05-04

**Authors:** Edgars Pudzis, Sanda Geipele, Armands Auzins, Andrejs Lazdins, Jevgenija Butnicka, Krista Krumina, Indra Ciuksa, Maris Kalinka, Una Krutova, Mark Grimitliht, Marii Prii-Pärn, Charlotta Björklund, Susanne Vävare, Johanna Hagström, Ingela Granqvist, Malin Josefina Hallor

**Affiliations:** 1 Institute of the Civil Engineering and Real Estate Economics, Riga Technical University, 1048 Riga, Latvia; edgars.pudzis@rtu.lv (E.P.); armands.auzins@rtu.lv (A.A.); andrejs.lazdins@rtu.lv (A.L.); maris.kalinka@rtu.lv (M.K.); una.krutova@rtu.lv (U.K.); 2 The Ministry of Environmental Protection and Regional Development, 1494 Riga, Latvia; jevgenija.butnicka@varam.gov.lv (J.B.); krista.krumina@varam.gov.lv (K.K.); indra.ciuksa@varam.gov.lv (I.C.); 3 Saaremaa Municipality, 93819 Kuressaare, Estonia; mark.grimitliht@saaremaavald.ee (M.G.); marii.prii-parn@saaremaavald.ee (M.P.-P.); 4 The Government of Åland, 22 100 Mariehamn, Finland; charlotta.bjorklund@regeringen.ax (C.B.); susanne.vavare@regeringen.ax (S.V.); 5 Mariehamn Municipality, 22 101 Mariehamn, Finland; johanna.hagstrom@mariehamn.ax; 6 Norrköping Municipality, 601 81 Norrköping, Sweden; ingela.granqvist@norrkoping.se (I.G.); malin.hallor@norrkoping.se (M.J.H.)

**Keywords:** formal planning, informal planning, spatial planning process, coastal area spatial planning, planning levels, community involvement, territorial community, coastal communities

## Abstract

Many shared views of both scholars and practitioners reflect spatial planning as a place-creating process that must be understood from a multi-level perspective. Formal and informal planning modes have variations in planning practices in different countries. In this study, we aimed to evaluate the interaction of formal and informal spatial planning in the frame of the spatial planning system in the Baltic Sea region. We were searching to highlight the involvement possibilities of territorial communities in the spatial planning process around the Baltic Sea region, focusing on coastal areas and their specific features in Latvia, Estonia, the Åland Islands of Finland, and Sweden. Involved experts expressed views based on a pre-developed model to identify how institutionalized formal spatial planning relates with informal interventions. This allowed the development and proposal of a model for coastal area spatial planning and implementation. We concluded that in the spatial planning approach, the governance works differently in different countries, and coastal area spatial planning differs from regular spatial planning. The information base is sufficient to initiate spatial planning at the municipal level, but municipalities should be more active, involving territorial communities in the planning, implementation, and control of municipal spatial planning, as this ensures a greater interest in the use of planning outcome.

## 1. Introduction

With increasing urbanization, particularly in the coastal areas of the Baltic Sea region, there are several sustainable development problems identified, e.g., environmental pollution, community living, and preservation of cultural and natural heritage. A comprehensive and local needs-based planning approach to the sustainable development of maritime and coastal areas in the Baltic Sea region is relevant and consequently better for decision-making by local and regional governments [1]. Therefore, the formal and informal spatial planning process of the coastal zone needs to be evaluated. To understand the importance and interrelationship of formal and informal planning, it is necessary to assess the stages of mobilization, planning, implementation, and monitoring (analysis structure).

An increasing proportion of the world’s population is based in coastal cities because of the beneficial circumstances and services they provide [2]. The Baltic Sea coast is attractive in many aspects. Interests from different sectors all have claims on the coastal areas resulting in major pressures on valuable marine habitats, natural resources, and ecosystem services. There is a joint requirement for improved management strategies to work with spatial planning in a more holistic, sustainable, and efficient way. In the frame of the Interreg Central Baltic project Coast4us in all participating countries (Latvia, Estonia, the Åland Islands of Finland, and Sweden), the problem is that many relevant interests, e.g., environmental, economic, social, or cultural, are not sufficiently recognized in the planning process. This non-holistic approach results in actions that are less viable and are less adapted to local interests, and thus they become less cost-efficient. The idea of the project Coast4us is therefore to work with a holistic approach in the planning process, involving stakeholders of different interests in a participative way, and create sustainable marine and coastal zone plans. The main objective of the project Coast4us is to create sustainable plans for marine and coastal areas [1].

To achieve faster territorial growth and to provide positive changes in regions and local municipalities, it is essential to involve wider society, including entrepreneurs, local communities, and citizen groups, in addressing territorial development issues. A formal planning approach refers to the provision of a formal planning process which is regulated by established institutional settings and implemented through a set hierarchy of competences, e.g., planning and governance levels. Formal planning also provides formal planning tools, e.g., a long-term comprehensive plan for the local government territory (municipality). In the context of planning, Syssner and Meijer [3] assume that formal planning processes contain elements of informality. The concept of formal planning can be used to refer to the kind of planning that is government-led and shaped mainly by formal structures and through formal negotiation. However, beyond the formal planning system, we may recognize some attempts of citizens to “adapt space” according to their needs.

Informal planning approaches, such as community planning and participatory budgeting, might help local governance better understand the needs of local society, especially in large municipalities also addressing the needs of peripheral areas. It might help to provide the best tailored solutions for different communities/areas. In addition, it might improve better societal trust in public governance. Furthermore, an informal planning approach might provide an opportunity for the society/active citizens to present their needs to the local governance, providing more fruitful dialogue with local governance and engaging in a public decision-making process. It might help them to better contribute to the development of living environment attracting necessary support from local governments and other parties, promote local patriotism and a sense of belonging to their place of life, as well as to improve their knowledge about the development planning and implementation processes. Local initiatives are of great importance as often the local community might address the challenges in the most efficient way. These assumptions have been discussed among participants of the project Coast4us during organized networking and workshops. However, it is necessary to explore how formal and informal spatial planning processes in the specific coastal conditions support the development and implementation of long-term strategies and plans, as well as how these processes interact with and influence the sustainability of local communities.

The scientific literature on the territorial community emphasized five core elements. First, locus, a sense of place, referred to a geographic entity ranging from neighbourhood to city size, or a particular milieu around which people gathered, such as, a church or recreation centre. Second, sharing, common interests and perspectives, referred to common interests and values that could cross geographic boundaries. Third, joint action, a sense of coherence and identity, included informal common activities such as sharing tasks and helping neighbours, but these were not necessarily intentionally designed to create community cohesion. Fourth, social ties involved relationships that created the ongoing sense of cohesion. Finally, a diversity referred not primarily to ethnic groupings, but to the social complexity within communities in which a multiplicity of communities co-existed [4]. We may see the territorial community as the common territory of existence, the presence of common interests of local importance, social interaction of community members in the process of ensuring these interests, psychological self-identification of each member with the community, common communal property, and payment of utility taxes [2]. Therefore, in the present study, the territorial community in Latvia is a village, in Estonia, an island, in Sweden, a village, camping site, and unexplored island, and in the Åland Islands of Finland, urban and rural areas [1].

The experts of participating countries in the project Coast4us addressed their views about the spatial planning system and process in their territorial community. **In Latvia**, the villagers have a better understanding of the situation on the ground and a better understanding of the needs of the inhabitants of the village than the people from outside of the village. The involvement of the population allows for better control of the result to be achieved. The villagers better maintain their objects because they are more interested. **In** **Estonia,** continuously involving and engaging different social groups in the early visioning and planning process should be a binding part of every development. **In Sweden**, it is important to listen to local experts and actors on site. Therefore, it is necessary to involve and engage them in an early stage for development. **In** **Åland**, it is important to get local people involved in an early stage in the planning process, to ensure people understand what needs to be implemented. The purpose of the whole process should also be described based on sustainability principles, because planning and a future sustainable society with a working green infrastructure must go hand in hand. We need to prevent the effects of climate change and we need to increase and strengthen biodiversity because it gives society a greater resilience.

The **hypothesis** of this study is that spatial planning systems in the Baltic Sea region are in the process of changing in order to involve society in the spatial development of their territory.

For this study, several **research questions** were developed. First, how can the spatial development planning process improve the life of local communities in the specific coastal conditions? Second, what is the significance of both formal and informal planning approaches, and the impact of their interaction? Third, what are the possibilities to involve in the coastal area spatial planning process for the population groups in territorial communities?

In order to answer the research questions, a multi-element study is needed, which includes: (1) theoretical aspects on the features of formal and informal planning; (2) an overview of special needs of coastal communities, comparison, and evaluation of the spatial planning process in specific coastal areas (considering that one coastal area consists of different countries with different planning systems); and, (3) the impact assessment of the stages of the spatial planning on meeting the special needs of the coast.

The multi-element study is being developed for the Baltic Sea coast, based on information from national experts, as well as the study of specific pilot areas around the Baltic Sea.

**The pilot areas of Latvia** are two small village communities from two different coastal local municipalities: (1) Tuja village in Salacgriva municipality, and (2) Garupe village in Carnikava municipality. **The pilot area in Estonia** is the largest island in Estonia-Saaremaa. This is the main island of Saare county and it belongs to the West Estonian Archipelago. Since the end of 2017, based on all twelve former municipalities, Saaremaa has been governed as one municipality. **The pilot areas on mainland Åland** are: (1) an urban area in the city of Mariehamn, and (2) a rural area in Sund municipality. **The pilot areas in Sweden** are from three different coastal local municipalities: (1) a small village in Arkösund/Norrköping municipality, (2) a camping site in Ekön/Valdemarsvik municipality, and (3) the unexplored island Bergön in Söderköping municipality.

**The purpose of the research** is to evaluate the interaction of formal and informal spatial planning processes in the coastal area through comparative study, considering the hierarchies of spatial planning systems in selected and differently experienced country cases around the Baltic Sea region. Accordingly, **the objectives** are set as follows: (1) to describe the theoretical aspects of the formal and informal planning; (2) to compare and evaluate the spatial planning process of Latvia, Estonia, the Åland Islands of Finland, and Sweden by levels of the spatial planning system; (3) to conduct an expert survey on the impact of formal and informal planning processes by using the stages of cooperation, i.e., mobilisation, planning, implementation, and monitoring; and (4) to propose a model supporting coastal area spatial planning and implementation for community involvement.

The research uses: (1) a literature review method for an overview of theoretical aspects of the formal and informal planning and a comparative evaluation of the planning process; (2) an expert assessment method employed by the experts from Latvia, Estonia, Finland, and Sweden for a description of the situation, according to the objectives of the project and the study, as well as to conduct an expert survey; and (3) discourse analysis and synthesis as well as graphical methods for designing main research results, including a proposed model. The collection and analysis of secondary and primary data are performed by the authors of the study. The expert survey in (2) is based on the methodology previously developed by researchers at Riga Technical University [5].

## 2. Theoretical Background

The theoretical aspects of formal and informal spatial planning are closely related to settlements, i.e., villages and towns. Historically, developed cities and other places are the largest complex adaptive systems in human culture and have always been changing over time according to largely unplanned patterns of development [6]. To a large extent, the development of processes is shaped by the potential for co-optation. Erikson [7] outlined four characteristics of collaboration: (1) the bonding between the parties, incorporating the user representatives in the organizations and their institutional logic; (2) the organizational framing of the user involvement activities, setting the initial rule for how to act/speak, where to act/speak, when to act/speak, as well as what to speak about; (3) the organizational control exercised as the activities took place, directing the discussions and interactions to align with the interests of the welfare organizations; and (4) the resistance exercised by user representatives, enabling them to influence the organizations and contribute to change. Successful collaboration is based on available resources. Two resource groups are important in the planning process, namely, human resources and organizational resources. Their significance primarily consists of two facets: first, that they utilize existing knowledge; and second, that they create legitimacy and feelings of pride and belonging in the local community [3,8]. Collaboration can take place in a formal, semi-formal, and informal way, which means that spatial planning also has these forms of collaboration. From the best-practice perspective, decision-making in spatial planning must be decentralized, and the tools of spatial planning must be less binding (which has been broadly practised in Switzerland, for instance) [9]. 

The idea of the informal organization was first introduced by Barnard [10]. He compared the informal organization to a clique or an exclusive group of people that naturally forms over time. According to Barnard, this can be achieved by linking Maslow A’s theory of needs and central ideas to new concepts of leadership [11]. Many scholars have studied the involvement of informal social groups in the planning processes, including spatial planning. For example, Certoma [12] defined five tools, evidence, knowledge, encouragement, evaluation, and assistance, which are related to the aim of the study. However, we must first distinguish between spatial planning systems and planning practices, the latter of which reflects the planning culture. Reimer, et al. [13], interpreted planning systems as “dynamic institutional technologies, which define corridors of action for planning practice, which may, however, nonetheless display a good deal of variability”. Fürst [14] equated the planning culture to the values, attitudes, mind-sets, and routines shared by those taking part in the planning process. Reimer, et al. [13] provided some arguments that planning practices inherent to the system cannot be drawn only from a comparison of legal–administrative framework conditions.

Comprehensive literature analysis allows for identifying the characteristics of informal spatial planning. Based on such an analysis, Mishra [9] formulated that informal planning should: facilitate the formal process; adds flexibility; contributes with matured results by discourses; is used as an ad hoc system when required; implies a degree of innovation, continuously meeting the planning challenges; is a non-traditional planning mode not influenced by the hierarchy culture of planning; is a multi-level collaboration process-oriented to consensus in decision-making; requires governance to avoid potential conflict and progress towards a legitimate solution; requires the inclusion of different stakeholders’ unbiased results or planning direction; and vitally needs transparency as it keeps the stakeholders of different levels well informed and avoids potential conflicts.

Following a “socio-institutionalism perspective”, this article refers to the assumptions guiding spatial planning and development. Therefore, the legal and administrative structures and competencies that shape possible spatial development or changes in land use (formal institutional settings) are introduced first to search for governance relations in planning and implementation processes. Institutions are established to organize a spatial planning process and provide measures for public involvement. Thus, the institutional performance refers to administrative structures, policy styles, institutional and social settings, collective actions, and social learning [15]. The planning process is concerned with deliberative plan-making, applying various planning modes of different scales, e.g., national, regional, local, and more detailed. However, the planning process may differ in terms of the extent of public involvement. This may be assessed, for instance, by analyzing the activities of stakeholders’ deliberation and informal population groups.

Formal and informal (complementary) spatial planning tools provide the necessary support to improve planning practices, but positively-influenced practices substantiate discourses (e.g., desirable dominating ideas) in spatial planning [15]. Informal planning tools often are developed as a result of using project-oriented techniques and integrated assessment tools, e.g., nature protection plans, management plans of water bodies, or assessments of ecosystem services. Relevant processes, e.g., formal and informal spatial planning, local development, and protection of valuable landscapes and related consequent decision-making, strive for collaborative learning by understanding the values of land-related resources and their most efficient usage. Auzins [15] provided main objectives for introducing a values-led planning approach as it promotes improved, more supportive, and collaborative territorial governance as well as informal institutions and organisational forms, as they significantly support formal spatial planning and social settings driven by common and local place-based interests.

At the same time, it is clear that spatial planning approaches and governance work differently in different countries. Indeed, even in Europe you can find varieties of approaches starting from integrated, regional-economic to just land-use management approaches. Coastal area spatial planning differs from regular spatial planning because it is connected with specific water objects (Baltic Sea in the case of this study). The water object is placed on territories of different countries with different legislation, history, and governance. With these circumstances, it is important to analyze planning conditions, and share knowledge to, from one perspective, sustainably use and develop a common resource (Baltic Sea in case of this study), and from another perspective, make equal opportunities for the development of coastal communities, regardless of political circumstances.

Spatial planning, implementation, and monitoring need to have a certain order to ensure common processes and results [3]. At the same time, for instance, the latest research in village and community planning in Latvia proposed that the methods of involvement must be as diverse as possible [16]. This clearly indicates that, as a result of societal growth, the planning process must be changeable and modern, which forces the research community to constantly look for new and appropriate opportunities for formal and informal societal involvement and motivation.

Community involvement has been shown to make a positive contribution to planning and development processes. At its best, community involvement can enable the following: processes to be sped up; resources to be used more effectively; product quality and feelings of local ownership to improve; added value to emerge; confidence and skills to increase for all; and conflicts to be more readily resolved. Public participation should be an indispensable element in human settlements, especially in planning strategies and in their formulation, implementation, and management. It should influence all levels of government in the decision-making process to further the political and economic growth of human settlements [17].

Recently, Geipele, et al. [18] highlighted several specific spatial planning problems of coastal communities in the Baltic Sea region by evaluating community involvement in participatory processes: (a) lack of communication, regardless of country and region; (b) the weak involvement of different social groups; (c) insufficient coastal and environmental management; (d) excessive regulatory enactments; and (e) summer-year-round population conflict. These problems must be taken into account, but have not been analysed in this study.

## 3. Materials and Methods

In general, qualitative research methodology (descriptive, logical, comparative expert assessment) and quantitative research methodology (factual comparison methods) are used for the study.

The experts, who have been involved for the development of the study, represent the local (municipal) and governmental authorities as well as scientific sectors to assess the formal and informal territorial coastal spatial planning process in Latvia, Estonia, the Finnish Åland Islands, and Sweden. An expert group consisting of four subgroups and representing particular countries has been established for the study: (1) in Latvia, three scientific experts and three experts from national-level authority; (2) in Estonia, two experts from municipal authority; (3) in the Åland Islands of Finland, two experts from the government and one expert from municipal authority; and in (4) Sweden, two experts from local, municipal authorities. The size of the expert subgroups was determined by the number of participants involved in the project (determined by the team leader).

For the overview, analysis, and discussion of spatial planning systems, the experts explored particular institutional settings, legal instruments, and policy planning documentation. The results are summarized in Table A1 (see Appendix A). The expert survey was conducted to assess the impact of formal and informal spatial planning on community development by using four stages of cooperation. From the preliminary networking in the frame of the Coast4us project, it was acknowledged that selected experts were professionally competent enough to deliver knowledge that is accumulated from formal and informal population groups of communities, and thus, represents the dominating opinion of local communities in specific pilot areas of the coast. Therefore, they gave some discursive influence on research, as they were largely in charge of relevant spatial planning and implementation processes. The designed structured expert survey consisted of two parts to particularly analyze the significance of both formal and informal planning. Each part included 12 questions to the respondents. The responses to these questions allowed scoring from 1 to 10 (from low significance to high significance). The average rating data demonstrated some interpreted trend and ground synergistic planning models. The Section 6 of the study displays summarized results and proposes the model for cooperation as a synthesized outcome.

## 4. Pilot Areas for Research

**Figure 1:**
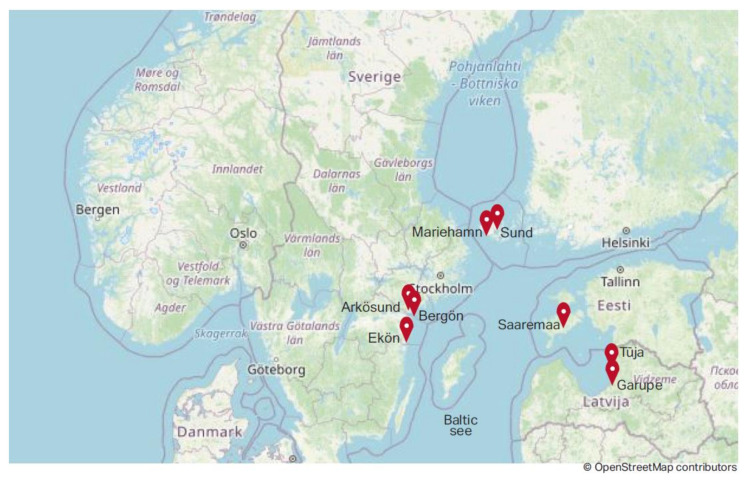
Pilot areas of the present study on the Baltic Sea region map.

Figure 1 shows marked pilot areas of this study in the Baltic Sea region.

### 4.1. The Case Study of Latvia: Description of the Villages

Garupe village is in the Piejura lowland, Carnikava municipality (Latvia) about 4 km to the southwest from the administrative centre of Carnikava, and 26 km from the centre of Riga. The village is located in the surrounding area of capital Riga. The environmental conditions in Garupe are largely determined by the fact that the village is in the territory of the Eimura-Mangali polder. The spatial structure of Garupe village is characterised by dense summer house/dwelling house construction, a drain system, and a dense street network. Garupe is a residential village. The population of Garupe is gradually transforming from a seasonal to a permanent population. On average, 405 people live in Garupe. During the summer, the total population reaches over 1000 people. People work in nearby areas, especially in Riga. Experts concluded that Garupe is a typical urban extension of the Riga city and the local community is forming at its stage of development.

Tuja village is a populated place in Liepupe parish, Salacgriva municipality, Latvia. It is located on the coast of the Gulf of Riga, on the banks of Zakupite, 33 km from the centre of Salacgriva district and 75 km from Riga. On average, 276 people live in Tuja. In Tuja, the territory of detached houses occupies 44%, recreation area—11%, territory of garden plots—2%, and mixed business area—5% (approximate distribution). There are two campsites and a beach in Tuja. There is a long fishing tradition and public services are available.

### 4.2. The Case Study of Estonia: Description of the Island

Saaremaa municipality is located on the largest island in Estonia-Saaremaa. It is located in the Baltic Sea, south of Hiiumaa island and west of Muhu island, and belongs to the West Estonian Archipelago. It is the fourth largest island in the Baltic Sea. Its territory is 2718 km^2^ and the shoreline is 874 km long. There are 4 large islands in Saaremaa municipality: Saaremaa, Abruka, Vilsandi, and Kõinastu and a number of smaller islands. In Saaremaa municipality, there is 1 town, Kuressaare, 9 boroughs, and 427 villages. There are 31,466 inhabitants (as of 1 April 2019), and the population is descending and ageing. Inhabitants work mostly in electronics, boat building, food, or the plastic industry, but also in agriculture, fishing, and tourism. Many people are self-employed, such as little holiday homeowners, craftsmen, small producers or small farmers, and many people do telework.

Because of its mild maritime climate and a variety of soils, Saaremaa has a rich flora, including rare orchid species, but also a wide variety of rare wildlife species, ranging from insects to seals. There are also a number of semi-natural communities (wooded meadows, alvars, floodplain meadows, and coastal meadows). All this biological richness is the reason why almost 18% of the Saaremaa community is under nature conservation. Beautiful nature, interesting geology, safe environment, unique cultural heritage, and many created facilities (spas, hotels, tourism farms) are the reasons why Saaremaa is a quite popular tourism destination. In addition, it is common to own a summer house there, to be used seasonally. Saaremaa can be visited by ferry or by plane, but there are also many small marinas for yachts or other small boats.

The aforementioned components need a comprehensive understanding and vision. In the project, the main aim is to generate valuable input material for the Saaremaa municipality, including a comprehensive plan regarding coastal area region.

### 4.3. The Case Study of the Åland Islands of Finland: Description of the City and Rural Area

Mariehamn city is the capital of the autonomous Åland Islands, where about 11,000 people (of the total 30,000 Åland population) live. Here, one may find shops, cafés, camping grounds, public beaches, green areas, hotels, conference centres, marinas, and football fields. Mariehamn city also has a big harbour and is an important tourist destination during the summer months. Mariehamn is situated in the south of the mainland on a peninsula, almost completely surrounded by water. It is important to make sure that the activities on land do not harm nature on land or at sea. Therefore, in the project, the aim is to try to incorporate the importance of a functioning green infrastructure in the planning of the city.

Sund municipality is an area where the agriculture dominates, and the challenge is to bring back natural biodiversity and stop nutrient run-off from land to sea.

### 4.4. The Case Study of Sweden: Description of the Village, Camping Site, and Unexplored Island 

Arkösund is in the eastern part of the Vikbolandet peninsula, 50 km from Norrköping city and 200 km south from Stockholm. The district is located in the Östergötland region territory.

Östergötland’s northern archipelago, with the bay of Bråviken, shores along the Vikbolandet peninsula, and the skerries and islands outside, is called Arkösund Archipelago. The Gränsö-Birkö-Aspö-Arkö island chain outside the channel by Arkösund is composed of larger islands with a fair amount of settlement, both in the form of permanent residences and weekend cottages. The large shallow areas here have proven to have great biodiversity and different types of environments. That is one reason that the county’s largest nature reserve, Bråviken Nature Reserve, with more than 9000 hectares of protected land and water, has been accorded the status of a marine nature reserve. Many people who visit Arkösund come by boat since the north–south shipping line from Stockholm passes right by here. Arkösund is a natural junction. The community itself is an old fishing village and a seaside resort. At the end of the 19th century and beginning of the 20th, Arkösund was a summer paradise for wealthy Norrköping residents, which is abundantly clear from the many large villas. A special “resort train” ran on a narrow-gauge railway to the area. There were picturesque small walking paths with footbridges out to the islets, the so-called “Bathing Islands”. During the period from March to October, it is much frequented and a centre of activities. Here, one may find marinas, a boatyard, a petrol station, shops, camping grounds, conference centres, pubs, and hotels with entertainment. Although the activities vary from year to year, Arkösund is a place where there is always life and movement.

## 5. Assessment of Different Spatial Planning Levels, Including Formal and Informal Spatial Planning in Latvia, Estonia, Åland Islands of Finland, and Sweden

### 5.1. Overview of Spatial Planning in Latvia

In Latvia, the overall development planning system is determined by the Development Planning System Law, while the Spatial Development Planning Law focuses on spatial development planning at all governance levels. To pay more attention to the involvement of various groups of the inhabitants in the development planning, regulation on the Procedure for Involvement of the General Public in the Development Planning Process was adopted in 2008 [19]. The purpose of the regulation is to foster an effective, open, inclusive, timely, and responsible involvement of the general public into the development planning process, thus increasing the quality of the planning process and ensuring the compliance of planning results with the needs and interests of the population. The regulations on municipal spatial development planning documents and on planning region spatial development planning documents stipulate the involvement of the public in the preparation of spatial development plans [20,21].

**National level**. According to the legislation, at the national level, the following spatial development planning documents shall be elaborated: the Sustainable Development Strategy for Latvia and the National Development Plan. The Sustainable Development Strategy for Latvia is hierarchically the highest national level long-term planning document, which sets out the long-term development priorities and the spatial development perspective of the state. It is followed by the medium-term planning document, the National Development Plan, which defines priorities for sectoral policies, territory development and tasks and activities to be implemented thereof, as well as sources of financing. [22].

Following the Sustainable Development Strategy and National Development Plan, sectoral policy development planning documents shall be elaborated for a medium-term. The Regional Policy Guidelines 2021–2027 is the main medium-term policy planning document, which determines the regional policy of Latvia and elaborates on the National Development Plan. It provides directions of action and tasks in the field of regional development [23]. Development of a maritime spatial plan and national long-term thematic plan for public infrastructure development in the coastal area was introduced in 2014 [24]. 

**Regional level**. At the regional level, the sustainable development strategy and the development program of a planning region (for each of the 5 planning regions in Latvia) shall be elaborated. The sustainable development strategy of a planning region is a long-term spatial development planning document, specifying the vision of the long-term development, strategic objectives, priorities of the planning region, and the spatial development perspective in written and graphic form. The development program of a planning region (medium-term) shall contain the current situation analysis, tendencies, and forecasts, as well as information regarding the developing process of the development program, and shall define mid-term priorities, the set of measures for the implementation thereof and the procedures for monitoring thereof [25].

**Local (municipal) level**. At the local governmental level, the following planning documents shall be elaborated: the sustainable development strategy, the development program, the spatial development plan (a comprehensive plan), a local plan (for particular areas), and a detailed plan (for specific development areas/sites). The sustainable development strategy of a local government defines the vision of the local government’s long-term development, strategic objectives, development priorities, and the spatial development perspective in written and graphic form.

A local government development program (medium-term) shall include the analysis of the current situation, tendencies and forecasts, as well as information regarding the developing process of the development program, and shall define mid-term priorities, the action and investment plan, the number of resources necessary for the implementation of the development program, and particular procedures for monitoring of the development program. A local government development program, which is developed in an integrated manner, is a prerequisite to attract support from the EU funds.

The spatial development plan of a local government may be detailed in a local plan. After the sustainable development strategy comes into effect in a local government, the spatial development plan may be amended in the local plan, insofar as the local plan is not in contradiction with the sustainable development strategy of the local government [26].

**Village (community) level**. The formal development planning system does not provide a framework for planning at the village/community level in Latvia. It is a voluntary-based process, which can be initiated by informal groups of citizens. Although there are several examples of elaborated village plans, their implementation so far was not successful, due to the lack of support, including from public governance. Nevertheless, to attract the funding for local initiatives from the EU Agriculture Fund for rural development (LEADER approach), local action groups (LAG) shall elaborate local action plans, which define and justify priorities and necessary changes in the territory covered by the LAGs. This process is regulated by the regulations of EU funds attraction.

### 5.2. Overview of Spatial Planning in Estonia

**National level**. The hierarchically highest national level long-term planning document is the national spatial plan Estonia 2030+. The national spatial plan is a strategic schematic document aiming to achieve the expedient utilisation of space on the scale of Estonia as a whole. The national spatial plan is being prepared for the entire territory of the country. It defines the policies and trends for sustainable and balanced national spatial development. The purpose of the plan is to obtain spatial bases, informed by the specific character of the environment, for shaping settlement, mobility, national engineering infrastructure, and regional development [27].

In 2017 the government of Estonia initiated a thematic national spatial plan for maritime areas. The Estonian maritime spatial plan is a tool for the long-term planning of the use of the sea, which balances the social, economic, cultural, and environmental interests and needs. Maritime spatial planning enables the determination of where and under what conditions the implementation of different human activities in the marine area is most reasonable. This is to ensure the economic benefits resulting from the exploitation of marine resources, as well as the value of the sea and coastal areas as socially and culturally important areas, keeping in mind that any human activity must be based on the achievement or maintenance of the good status of the marine environment [27,28].

**Regional level**. Regional development planning is directed by the Regional Development Strategy 2014–2020. The key place in the regional development in Estonia is held by the development of centres and making better use of regional differences [29]. Following the Regional Development Strategy, the state is working to ensure consistent growth in all areas, applying the unique potential available due to each area’s peculiarities. The Estonian government ratified this strategy and its implementation plan for 2014–2020 in 2014. The strategy focuses on the development needs of all Estonia’s regions. The government is investing more than previously in the improvement of work availability and services in areas, which have been adversely affected by urbanization, by emphasizing the strengths and unique aspects of each region.

Main strategic goals are divided into four major groups:

- An environment for households and enterprises in the active regions, which supports their wholeness and competitiveness. To shape a balance regarding the draw of larger urban centres with stronger active regions across Estonia, having improved environments for living and entrepreneurship as well as diverse work service, and activity opportunities.
- An environment in major cities that promotes competitiveness in the international economy. To increase the importance of urban areas as centres of growth for an innovative and science-intensive economy with the help of an increasingly attractive living environment.
- Exploiting region-specific resources with greater skill. This promotes specialization in growing areas of competence and enterprise according to region-specific conditions, and increases clarity in the uniqueness of different areas.
- Greater connectedness and ability to grow. For regions to achieve a stronger ability to develop by greater inter-regional connectivity and increasing efficiency in regional cooperation and capacity for growth.

Regional (national and local as well) development planning is regulated by the Planning Act (PLA) [30]. PLA aims to create, through spatial planning, by promoting environmentally sound and economically, culturally, and socially sustainable development. These are the preconditions that are necessary for democratic, long-term, and balanced spatial development that takes into account the needs and interests of all members of the Estonian society to occur.

For each county, a regional development plan is implemented [31]. A county-wide spatial plan aims to define the principles and directions of the spatial development of the entire county, or a part thereof, or another region. A county-wide spatial plan is prepared primarily to express cross-border interests and to balance national and local needs and interests regarding spatial development. County-wide spatial plans are the basis for the preparation of comprehensive plans.

For example, Saaremaa County Plan 2030+ was implemented by the Minister of Public Administration in 2018. The main purpose of it is to balance the national and local interests, consider the local situation as well as to support the county’s spatial development, and ensure balanced and sustainable settlement structure and quality of life in the situation where the population of the county is shrinking and ageing [31].

**Local (municipal) level**. The functions and competence of a local authority include the organisation of spatial planning in the rural municipality or city. The instrument that directs spatial strategic planning and spatial development in the local municipality is a comprehensive plan. It is mandatory for every municipality. A comprehensive plan aims to define the principles of and directions in the spatial development of the entire territory of a rural municipality or city or a part of such territory.

The PLA sets some functions of a comprehensive plan, such as: to specify the conditions directing the development of human settlement; to define the boundaries of areas of repeated flooding on the coastline; to set the high water marks of internal bodies of water with an extensive flooding area; to specify the conditions to ensure the functioning of the green network and to determine the restrictions resulting from such network; to state the conditions of public access to shore paths; and to extend or reduce the building exclusion zone of the shore or bank. The functions to be fulfilled by a comprehensive plan are decided following the spatial needs of the local authority and the purpose of the plan. For example, Saaremaa municipality currently has 22 comprehensive plans in force. In 2018, Saaremaa’s local council initiated by a resolution the preparation of a new comprehensive plan of the entire territory of the new municipality [32].

To implement the comprehensive plan and to create an inclusive spatial solution for the planning area, a detailed spatial plan is prepared to plan construction works. The detailed spatial plan grounds initialization of construction works.

**Village (community) level**. In Estonia, there is no formal village and community planning stage or practice. The level of a municipality (local government level) involves village planning. The comprehensive planning process gives possibilities to plan and work on different levels, including village visions. This is a good way to connect visions of different levels. Formally possible ways may not be the best solution, because informal ways may be more attractive and efficient for locals to negotiate and agree on village visions.

### 5.3. Overview of Spatial Planning in the Åland Islands of Finland

The Åland Islands have an autonomous governance model, which means that they have their own legal rules on some issues and all planning processes are formal. 

**National level**. Åland has a law called “Plan-och bygglagen” [33] (the Planning and Building Act). The purpose of this law is to regulate land use and construction, so that: the conditions for a good living environment are created and preserved; an ecologically, economically, socially, and culturally sustainable development is promoted; and cultural-historical values are preserved.

With planning and building permits matters, the following must be observed: the provisions of the Act on nature conservation; the Act on forest management; the Act on environmental impact assessment and environmental assessment; the Act on environmental protection; the Act on the protection of culturally valuable historical buildings; the Act on ancient monuments; the Act on the protection of the maritime cultural heritage; the Act on the application to the Åland Health Protection; and the Water Act for the Åland.

**Local (municipal) level**. It is a municipal matter following the Planning and Building Act to decide the planning of the use of land and water. Each municipality must have a current municipal overview that covers the entire territory of the municipality. The municipal overview shall indicate the direction for the long-term development of the physical environment and guide decisions on the use of land and water areas and how these will be changed and preserved.

The regulation on land use and development within the municipality is provided through general and detail plans. The general plan sets out the main features of land use in the entire municipality or part of it. The detailed plan specifies how a limited area of land in the municipality should be used and built.

When the draft plan is drawn up, Åland municipality and other bodies, legal entities, and persons affected by the planning process are able to consult and express themselves in writing or orally. The purpose of the hearing is to improve the decision basis and provide opportunities for transparency and influence. The submitted proposals are considered, and the results are communicated to the public (stakeholders). Before a plan is adopted, the municipality must “exhibit” the proposal for at least 30 days. Municipal members and others have the right to submit comments on the plan proposal in writing during the exhibition period.

The municipality handles the planning, controls, and supervision of construction in its territory. If necessary, the government of Åland can decide on land use for certain important social functions or for certain purposes that are of great importance to the society, e.g., traffic networks, harbours and airports, energy production, and waste management.

The level of a municipality involves village planning. There is no expert opinion on the level of village planning. Through the Coast4us project, we have learned how important it is to involve local people and their knowledge as well as municipalities and other local operators at an early stage in various planning processes and decisions. This is to increase the understanding of the population on how to plan the coastal zone in the long-term and in a sustainable way.

### 5.4. Overview of Spatial Planning in Sweden

**National level**. Swedish planning processes are influenced by EU directives, especially legislation concerning environmental issues. There are several laws and regulations on how one can build and shape the Swedish environment (in all aspects). There are plans for municipalities and regions, but there is no national planning for the entire territory of Sweden. However, the state can affect the plans of municipalities and regions with national goals and interests. Based on the Planning and Building Act, the county administrative boards should make sure that national targets are realised and that everyone considers the national interests which exist. The actions of county administrative boards can have a positive or negative impact, including on the health and safety of people, and the risk of accidents or floods.

**Regional level**. Regional planning works with larger areas than municipalities. Regions coordinate planning across municipal borders. These plans exist so that each region can develop based on its circumstances.

**Local (municipal) level**. The municipality works with physical planning. Physical planning is about how to use land and water areas, where buildings and roads should be located and how they should be designed. The municipalities follow the Planning and Building Act. There are three types of municipal physical plans: structure plans, detailed development plans, and special area regulations. The structure plan should cover the entire municipality’s area. It displays how the municipality would like the city and land to be in the future and which areas the municipality thinks should and should not be used for building purposes. Detailed development plans have rules for where new buildings may be located and how they should appear. Special area regulations are based on the structure plans and detailed development plans. For instance, they may ground the decisions about the territories of holiday houses [34]. Through the networking in Coust4us project, cooperation with the colleagues from Norrköping municipality was established. They contacted local associations (informal community groups). In cooperation, it was possible to listen and learn how to comply with the Planning and Construction Law, and to use the planning process to create better products in the long run.

When analyzing the spatial planning systems of different countries, it can be concluded that the governance of spatial planning in the explored countries of the Baltic Sea region each work differently. However, spatial planning in general and the planning systems are similar, as they include the national, regional, and local government levels. No country has a formal level of planning in the village or community. In all countries, the most direct and closest public involvement in the spatial planning process is at the local government level. Local governments are responsible for both sustainable spatial development planning and land use planning.

## 6. Comparison and Evaluation of Formal and Informal Spatial Planning Process in Latvia, Estonia, Åland Islands of Finland, and Sweden

In this section, the authors have used information and conclusions from parallel and previous research conducted by individual study authors on the phases [5] and methods [18] of public involvement in spatial development.

According to the assessment of different spatial planning levels in Latvia, Estonia, Åland Islands of Finland, and Sweden, the involved experts prepared the reports. They summarized the results on mobilization, planning, implementation, and monitoring ranked by the municipal role in planning, strengths, and weaknesses (see Table A1 in Appendix A).

Comparing the reports by the Latvian, Estonian, Finnish, and Swedish experts, there is evidence that the spatial planning process is associated with a hierarchical structure. Long-term development documents at national, regional, and municipal levels (keywords: sustainability, efficiency, resources) provide the main guidelines for site maintenance and use.

When analysing the involvement of local community (specific coastal territories in each country case) at lowest and closest level for an individual, it has been concluded that at the beginning of the planning process (mobilization), municipalities in all participating countries invite citizens and stakeholders (informal groups) to get involved in the planning process due to the dissemination of information and discussions. All countries’ experts emphasized that the information is sufficient for the initial planning process. The weakness of the initial planning phase lies in the lack of communication with informal population groups.

In the spatial planning process, the local authorities have an information base (legislation, statistics, reports, opinions) to carry out the planning work. Experts emphasized that there is insufficient information about specific places and objects, and their functionality. There is information that in Åland, the autonomy function allows for extensive use of information. Informal groups do not participate in the planning process (document preparation), but they participate in the discussion of plans that have already been developed before they enter into force.

The plan is implemented by the municipality, following the developed plan, and granted funding. Both the plan as a planning tool and the implementation process are public. As a weakness, experts mentioned the impact of external factors that can change the course of the project, including various communication barriers that can cause controversy. Experts did not mention the role of informal groups in the implementation phase of the plan.

During the control phase, the municipalities monitor the implementation of the plan and provide reports following the established regulatory framework. At this stage, the availability and operability of the information are important factors. Experts mentioned the process was difficult to monitor, but did not mention the role of informal groups in the control process.

To better understand the benefits and challenging issues of the project Coast4us implementation process, an expert survey of responsible persons involved in the project was conducted. The essence of this survey was to assess the impact of the formal and informal spatial planning process in the specific coastal conditions, considering community involvement. The significance scale from 1 (insignificant) to 10 (significant) was used in the survey.

**Figure 2:**
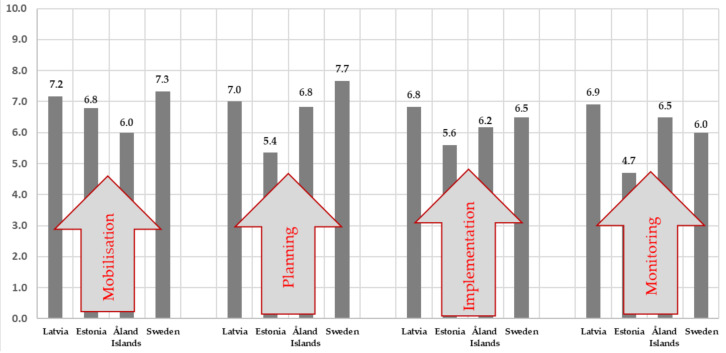
The summary of survey significance indicators (formal and informal spatial planning process).

Significance averages range from 4.7 to 7.7, which is quite wide. In the mobilization phase, the significance indicators are closer (6.0–7.3), which can be explained by the great importance of village development and sustainability. At the planning stage, there is a larger range of indicators of significance (5.4–7.7), which can be explained by the ambiguity of the goal definition and planning process (the coordination of opinions). The averages of the significance of the implementation phase are slightly scattered (5.6–6.8), which can be explained by the compromise reached in the planning process, but more in-depth research would be needed. In the monitoring phase, which is closely related to the implementation phase, the significance indicators of the obtained results are scattered (4.7–6.9), which can be explained by the evaluation of the process and the result (see Figure 2).

Based on the expert evaluation summary of the spatial planning process (mobilization, planning, implementation, and monitoring) and the answers provided by experts, the theoretical models of the functioning of informal organizations, as well as the results of expert discussions, a model has been developed by all research authors.

**Figure 3:**
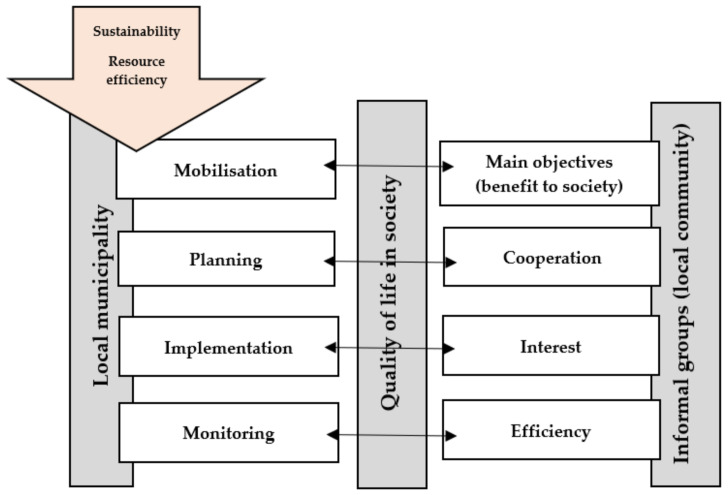
Model of cooperation between municipalities and territorial communities in the spatial development planning process.

The results of the study show that mobilisation, planning, implementation, and monitoring binds to Eriksson’s model of collaboration [7]. A successful project is based on a concerted goal that is in the interest of the parties involved (central government, local government, and informal groups). Collaboration in planning is essential to achieve the goal, as citizens are often more aware of the situation and they will be the ones who will use the results. Stakeholders’ participation in project implementation should be ensured as it avoids conflict situations, but monitoring ensures more efficient use of resources and a result of much higher quality. The members of local communities can be mobilized if their goals are clear, and these goals meet their interests. The spatial planning process should be open, involving and listening to the local community, which in this study were coastal communities. In the process of implementing the plan, the local community must be interested, and it will bring good results (and aid in future cooperation). A good result is achieved by monitoring the progress of the project and efficient use of resources, and all of this is confirmed by the informal groups (see Figure 3).

In addition to the aforementioned and conducted comparative research, there is a clear need to comply with the national regulatory framework in the process of community engagement while creating new and modern solutions for informal and motivated public involvement in the spatial planning process. At the same time, the study exploring different countries with the unifying object of the Baltic Sea, highlights the specifics of coastal area spatial planning. Therefore, regarding the usual sustainability factors, particular attention should be paid to natural and environmental values, conservation, and development of ecosystems, etc. These specific features, as well as the changing and rapid growth of society in the direction of community development, offer new directions of research for both academics and scientists.

## 7. Conclusions

The hypothesis of this study is confirmed because there is evidence that spatial planning systems are in the process of transformation and in fact “approach” the local population, as the focus of development shifts to the needs of a particular person in a specific place (local community) using new informal methods.

This study is a result of an extensive and in-depth collaboration between participants from different countries and spatial planning traditions of the Baltic Sea region, but with a common interest in formal and informal spatial planning processes in coastal areas. 

According to research questions mentioned in the introduction of this study and the multi-element research conducted, it can be concluded:

- both similarities and differences of spatial planning approaches have been detected in different countries that are placed around one water object, namely the Baltic Sea. All countries that have been analyzed have hierarchical planning systems and historically have used formal “top-down” spatial planning approaches. In recent years, spatial planning systems are changing to more “bottom-up” systems, but each country is conducting these processes in different ways. This makes risks to sustainable governance of the Baltic Sea and coastal communities;
- in all countries standard spatial planning process steps (mobilization, planning, implementation, and monitoring) are done, but it is clear that when a new “bottom-up” system is implemented, informal and more community-involved activities are done in step mobilization and planning. However, steps implementation and monitoring is mostly done by municipalities and civil servants. This, by opinion of the researcher group, leads to situations in which the community loses interest in being active inhabitants, because local community can not affect real implementation of their ideas and needs. Besides, they are not a part of change management of sustainable development. This creates a large risk of local conflicts as well as causing loss of interest of local development.

It is important to take into account the condition that coastal territory planning has very specific circumstances, and is connected with one water object that does not have physical borders, but sustainable development of these territories is very connected with this main resource (Baltic Sea). Mostly, development problems are similar, but they can be solved only when actions around the water object are equivalent. From our point of view, when different countries have mostly hierarchical planning structures, it is important that there are international agreements about main sustainable actions that are supplemented with “bottom up” solutions at the local community level, and at the same time experiences of local solution best practices are shared around coastal areas of one water object with the goal of sustainability and harmonization of actions.

The authors of this study propose to authorities of the spatial planning process for coastal areas around the Baltic Sea region to create a system, model, or even regulation, to make local (coastal) communities a part of the planning system at all phases (including implementation phase). This would reach such goals as: sustainable development, a “bottom-up” approach, and active citizenship. In addition, there is a proposal for better communication between countries, municipalities, and local communities around one water object to share best practices and harmonize actions.

## Data Availability

Not applicable.

## Appendix A

**Table A1:** Spatial planning process steps (mobilization, planning, implementation, and monitoring).

Country	Stage	Strengths	Weaknesses
Mobilisation			
**Latvia**	The local government invites the village representatives to participate in the planning process, disseminate information in a formal way (website, newspaper, etc.).	Widely available resources for the dissemination of information. Available communication contacts with citizens.	There is no direct daily contact with potential participants. No traditions yet.
**Saaremaa municipality Estonia**	Invites interest groups to participate in the planning process. Information is disseminated in a formal way (website, newspaper, exchange of formal letters, social media, etc).	Widely available resources for the dissemination of information.	The report does not identify any weaknesses.
**Åland Islands of Finland**	All interested parties shall be allowed to consult and make their views known in writing or orally where this is appropriate for the purpose and relevant to the planning process.	There is communication with citizens and local organizations.	The locals should be involved at an earlier stage before a proposal is made, communication should begin at an earlier stage.
**Sweden**	All stakeholders shall be allowed to consult and make their views known, in writing or orally, as appropriate to the purpose and relevant to the planning process. There is no strict national strategy for spatial planning.	There is communication with citizens and local organizations, and national interests are respected.	The report does not identify any weaknesses.
**Planning**			
**Latvia**	The local government, based on statistical research, modern solution identification and clustering of working groups, prepares the documents that are publicly discussed and approved within the framework of laws and regulations.	Resources are available for extensive statistical research. Resources are available for research in various industries, attracting both local and foreign specialists. Possible exchange of experience by using gathered contacts.	The use of formalised procedures. The lack of knowledge about specific problems in a specific place. Formal discussion of documents, as well as reliance on the laws and regulations that in the case of Latvia are often fragmented, or there is no available funding or other resources to introduce the regulations.
**Saaremaa municipality Estonia**	The local government, based on research, knowledge from previous plans, strategies, and information from different involved parties (interest groups, governmental institutions, etc.) prepares the documents that are publicly discussed and approved within the framework of laws and regulations.	Resources are available for extensive statistical research. Possible exchange of experience by using gathered contacts.	Insufficient knowledge about specific problems or possibilities in a specific place.
**Åland Islands of Finland**	Planning is formal, the island has its legislation, but the planning process follows general national legal norms.	The Government of Åland and other municipalities, authorities, legal persons, and persons shall be allowed to consult and make their views known, in writing or orally, as appropriate to the purpose and relevance of the plan. The purpose of the hearing is to improve the basis for decisions and to ensure transparency and influence.	Municipal members and others have the right to submit written comments on the plan proposal during the exhibition. There is limited time for collaboration in planning.
**Sweden**	Planning must respect national interests, EU law (special environmental issues).	All stakeholders are involved in the planning.	There is no national planning for the entirety of Sweden.
**Implementation**			
**Latvia**	Following the approved action plan and investment plan, the local government ensures the implementation of activities.	There are public resources available for the implementation of activities. There are tangible and intangible assets available to ensure the place for the implementation of activities.	Restrictions on the activities directed towards the regulation of statutory acts. Not being “on the site” ** makes it difficult to identify the most effective solution.
**Saaremaa municipality Estonia**	Following the approved strategic and funding plan, the local government ensures the implementation of the planning document and its principles.	The adopted planning document is public. Principles directing spatial planning are available online.	Planning document may not be flexible enough as there may occur changes in laws, unpredictable developments, etc. (not being up to date). Adopted principles may be understood differently (e.g., because of the wording).
**Åland Islands of Finland**	According to plan.	About the results of the hearing and proposals based on the different opinions that have been made shall be reported when the plan proposal is presented for the public.	The report does not identify any weaknesses.
**Sweden**	The planning process and implementation are regional and local in nature.	A better understanding of local conditions, fewer errors in implementation.	The report does not identify any weaknesses.
**Monitoring**			
**Latvia**	Following the regulations, the local government draws up regular reports on the progress, informs the Council about the reports, and publishes the reports online.	The data are made available, and there are restricted access databases. There are specialists to perform the work, and the necessary capacity is ensured.	A difficult-to-manage and slow (change) management process. Formal reports based on data collection.
**Saaremaa municipality Estonia**	Following the Planning Act, the local municipality needs to follow, review, and implement adopted spatial plans.	The planning process is public. There are specialists to perform the work, and the necessary capacity is ensured.	The report does not identify any weaknesses.
**Åland Islands of Finland**	The municipality handles the planning, controls, and supervision of construction within the municipality.	The report does not identify any strengths.	The municipality handles the planning, controls, and supervision of construction within the municipality.
**Sweden**	Monitoring shall be provided at all levels, following national interests and regulatory requirements.	The planning process is public. There are specialists to perform the work, and the necessary capacity is ensured.	It is indistinct who has the legal right to appeal the detailed development plans. This confuses and prolongs the process.

** Not being “on the site” – not living in a community on a daily basis, lack of knowledge of the community daily processes.
